# Intrinsic nanofilament pathways in molecular crystals enable energy-efficient and reliable memristors

**DOI:** 10.1093/nsr/nwaf482

**Published:** 2025-11-04

**Authors:** Yichun Liu

**Affiliations:** State Key Laboratory of Integrated Optoelectronics, School of Physics, Northeast Normal University, China

Memristors are widely regarded as promising building blocks for neuromorphic computing and in-memory processing, as they integrate memory and computation within a single unit, thereby reducing data transfer, improving energy efficiency and enabling real-time processing for artificial intelligence and dynamic vision applications [1]. However, practical implementation has been limited by intrinsic material challenges [2]. Conventional memristors based on atomic crystals, including transition metal oxides like HfO_2_ and layered 2D materials such as hexagonal boron nitride (h-BN), rely on ion migration through structural defects like grain boundaries, which introduces a series of drawbacks [3,4]. On the one hand, the stochastic migration of ions along grain boundaries leads to uncontrolled filament growth, resulting in elevated switching energies, accelerating material degradation. On the other hand, the inherent randomness of grain boundary distribution becomes increasingly detrimental as device dimensions are scaled down, giving rise to significant variations in switching behavior across devices. Such variability compromises device uniformity and dramatically reduces yield in large crossbar arrays, thereby constraining the scalability of atomic-crystal-based memristors [5]. Taken together, these limitations not only hinder intrinsic performance but also impose severe barriers to their practical deployment in integrated neuromorphic hardware systems.

**Figure 1.**
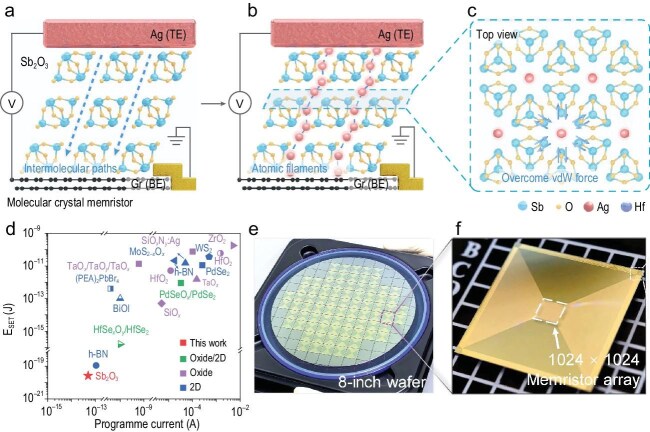
(a) Diagram of molecular crystal memristor in its pristine state before filament formation. (b) Illustration showing how the intermolecular spaces between Sb_4_O_6_ cages provide natural pathways for the growth of conductive filaments. (c) Top-view model of monolayer Sb_2_O_3_ along the (111) plane, indicating Ag ion migration through van der Waals gaps. (d) Comparison of the switching energy of an Sb_2_O_3_ memristor against recently reported high-performance devices. (e) Photograph of the fabricated 8-inch wafer. (f) Enlarged image of an individual die containing a 1024 × 1024 crossbar array. Reproduced from Ref. [6] with permission.

In a recent study published in *Nature Nanotechnology*, Zhai *et al.* report a novel type of memristor using a molecular crystal (Sb_2_O_3_) as the switching layer, which simultaneously addresses materials and integration challenges [6]. Sb_2_O_3_, composed of Sb_4_O_6_ molecular cages linked via van der Waals forces, provides natural low-energy pathways for Ag ion migration, enabling the controlled formation of uniform atomic-chain filaments while preserving lattice integrity during repeated switching (Fig. 1a–c). As a result, the devices not only exhibit ultralow switching energy of ∼26 zJ (Fig. 1d), but also exhibit remarkable endurance, sustaining stable resistive switching through more than 10^9^ cycles without any detectable degradation. Furthermore, as an inorganic molecular crystal, Sb_2_O_3_ can be deposited by standard thermal evaporation. This characteristic makes Sb_2_O_3_ particularly suitable for wafer-scale fabrication, and the authors have demonstrated 8-inch 1024 × 1024 crossbar arrays exhibiting 100% yield (Fig. 1e and f). Such compatibility with industrial semiconductor processes also supports the realization of integrated 1T1R arrays. These advantages highlight the excellent reliability of Sb_2_O_3_ molecular crystal memristors, making them a strong candidate for practical neuromorphic hardware.

In conclusion, this study represents a significant leap in memristor technology by introducing molecular crystal-based switching as a new paradigm for reliable, energy-efficient and highly scalable memory devices. Through the use of Sb_2_O_3_ molecular crystals with intrinsic van der Waals ion migration pathways, the authors overcome key limitations of conventional atomic-crystal-based memristors. The resulting devices demonstrate record-low energy consumption, exceptional endurance and excellent wafer-scale uniformity. These breakthroughs not only enhance device-level performance but also ensure compatibility with standard semiconductor fabrication, offering a practical and promising route toward integrated neuromorphic and in-memory computing hardware.
